# Oxidative stress and inflammatory markers are determinants of carotid artery disease quantified by magnetic resonance imaging

**DOI:** 10.1186/1532-429X-11-S1-P97

**Published:** 2009-01-28

**Authors:** Saurabh S Dhawan, Asad Ghafoor, Hamid S Syed, Christina Niessner, Konstantinos Aznaouridis, Muhammad Ali, Ibhar Al Mheid, Irina Uphoff, Charles B Kitchen, John Oshinski, Dean P Jones, Arshed A Quyyumi

**Affiliations:** Emory University School Of Medicine, Atlanta, GA USA

**Keywords:** Magnetic Resonance Image, Oxidative Stress, Thiol, Wall Thickness, Fibrinogen

## Introduction

Magnetic Resonance Imaging (MRI) can be used to measure common carotid artery maximum wall thickness (CCA-CWT) that incorporates the adventitia to intima-media thickness (IMT). Whether serologic markers of oxidative stress or inflammation are better predictors of wall thickness than conventional risk factors is unknown.

## Purpose

We hypothesized that patients with greater CCA-CWT have higher systemic levels of oxidative stress and inflammation.

## Methods

CCA-CWT was measured using MRI based T2-weighted black-blood sequence on transaxial slices in 92 subjects (61 ± 9 years) with IMT > 0.65 mm. Markers of oxidative stress included serum glutathione (GSH) and cystine (CySS), which are measures of reduced and oxidized thiols, respectively. Markers of inflammation included high-sensitivity c-reactive protein (HsCRP), tumor necrosis factor-alpha (TNF-α), monocyte chemoattractant protein-1 (MCP-1) and fibrinogen.

## Results

**Figure 1:**
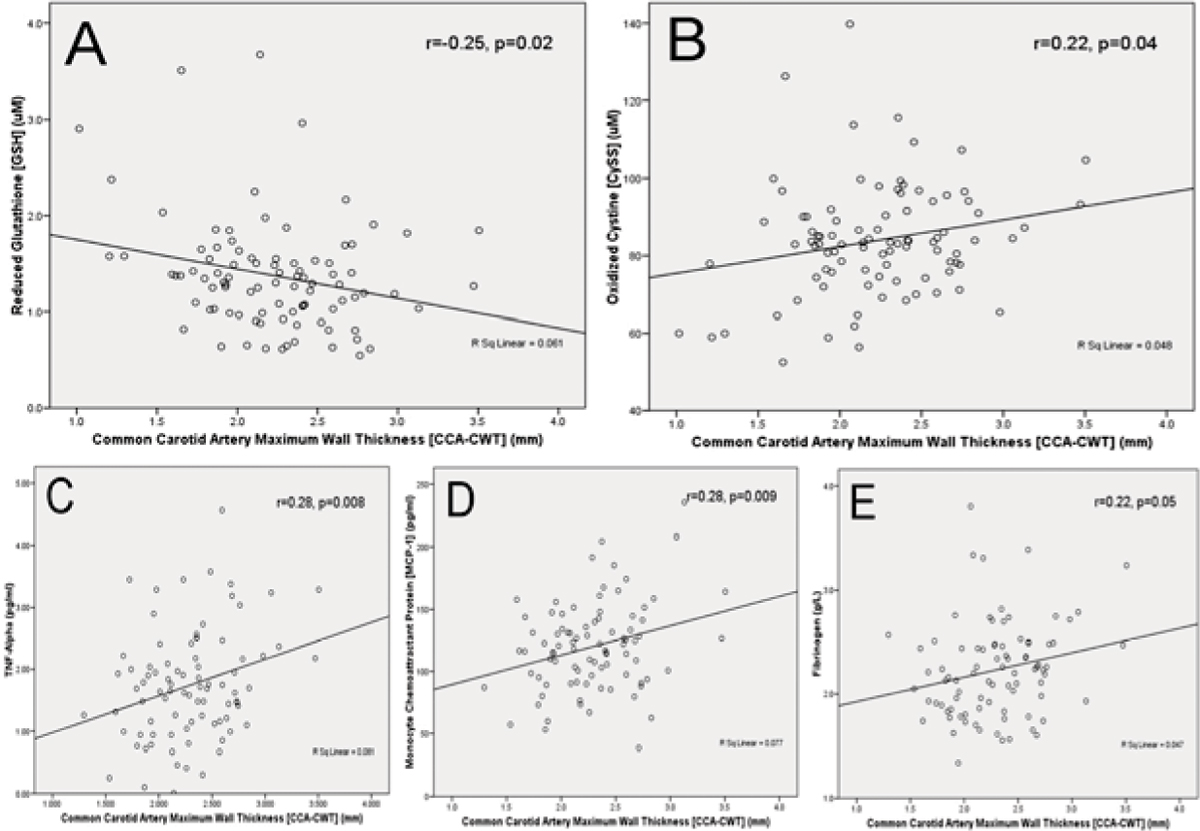
Figure 1

CCA-CWT correlated negatively with GSH (r = -0.25, p = 0.02) [Figure 1A] and positively with CySS (r = 0.22, p = 0.04) [Figure 1B], suggesting that oxidative stress was higher in those with more severe thickness. CCA-CWT also correlated with TNF-α (r = 0.28, p = 0.008) [Figure 1C], MCP-1 (r = 0.28, p = 0.009) [Figure 1D] and fibrinogen (r = 0.22, p = 0.05) [Figure 1E], but not HsCRP. These associations were independent of age, gender, BMI, hypertension, hyperlipidemia, diabetes and smoking.

## Conclusion

Biomarkers of oxidative stress and inflammation are better predictors of MRI quantified carotid artery disease than conventional risk factors. Whether progression of disease will also be predicted by these markers needs further study.

